# Activated carbons derived from coconut shells as high energy density cathode material for Li-ion capacitors

**DOI:** 10.1038/srep03002

**Published:** 2013-10-21

**Authors:** Akshay Jain, Vanchiappan Aravindan, Sundaramurthy Jayaraman, Palaniswamy Suresh Kumar, Rajasekhar Balasubramanian, Seeram Ramakrishna, Srinivasan Madhavi, M. P. Srinivasan

**Affiliations:** 1 Department of Chemical and Biomolecular Engineering, National University of Singapore, Singapore 117576; 2 Energy Research Institute @ NTU (ERI@N), Nanyang Technological University, Singapore 637553; 3 Department of Mechanical Engineering, National University of Singapore, Singapore 117576; 4 Department of Civil and Environmental Engineering, National University of Singapore, Singapore 117576; 5 School of Materials Science and Engineering, Nanyang Technological University, Singapore 639798; 6 TUM-CREATE, 1 CREATE Way, #10-02 CREATE Tower, Singapore 138602

## Abstract

In this manuscript, a dramatic increase in the energy density of ~ 69 Wh kg^−1^ and an extraordinary cycleability ~ 2000 cycles of the Li-ion hybrid electrochemical capacitors (Li-HEC) is achieved by employing tailored activated carbon (AC) of ~ 60% mesoporosity derived from coconut shells (CS). The AC is obtained by both physical and chemical hydrothermal carbonization activation process, and compared to the commercial AC powders (CAC) in terms of the supercapacitance performance in single electrode configuration *vs.* Li. The Li-HEC is fabricated with commercially available Li_4_Ti_5_O_12_ anode and the coconut shell derived AC as cathode in non-aqueous medium. The present research provides a new routine for the development of high energy density Li-HEC that employs a mesoporous carbonaceous electrode derived from bio-mass precursors.

The Li-ion hybrid electrochemical capacitor (Li-HEC) is one of the ubiquitous energy storage systems in this era^1^,^2^,^3^,^4^,^5^,^6^. Li-HEC exhibits the combined advantages of both lithium-ion batteries (LIB) and supercapacitors by delivering higher power and energy density than former and latter configurations, respectively. High energy and power density electrochemical energy storage systems such as Li-HEC are anticipated to drive zero emission transportation applications especially electric (EV) and plug-in/hybrid electric vehicles (PHEV or HEV) in the near future^7^,^8^. Generally, Li-HEC is constructed with the LIB component introduced as an insertion type electrode and high surface area carbonaceous materials as supercapacitor component in either aqueous or non-aqueous medium^1^,^5^. However, the development of higher energy density aqueous Li-HEC is highly restricted due to the water splitting issues of the electrolyte (~1.23 V). Therefore the concept of utilizing non-aqueous electrolytes in Li-HEC configuration has emerged and has been demonstrated using Li_4_Ti_5_O_12_ as Li-insertion type anode and activated carbon (AC) as cathode^2^. The configuration delivers the practical energy density of ~ 12 Wh kg^−1^ with reasonable power density^1^. Since then, several Li-insertion type electrodes such as LiNi_0.5_Mn_1.5_O_4_^9^,^10^, LiMn_2_O_4_^11^, LiFePO_4_^12^, LiCoPO_4_^13^, Li_2_MnSiO_4_^14^, Li_2_FeSiO_4_^15^, MnO_2_^16^, V_2_O_5_^17^, β-FeOOH^18^, Li_4_Ti_5_O_12_^1^,^2^,^19^,^20^,^21^, TiO_2_-B^22^,^23^, TiP_2_O_7_^24^, LiTi_2_(PO_4_)_3_^25^, and LiCrTiO_4_^26^,^27^ have been explored along with high surface area carbonaceous material as counter electrode. Among the insertion type electrodes proposed, spinel phase Li_4_Ti_5_O_12_ is found noteworthy due to its salient features such as (i) very high columbic efficiency (>95% at high current rates) with practical capacity close to the theoretical capacity of 175 mAh g^−1^ for reversible insertion of three moles of Li, (ii) thermodynamically flat discharge profile at ~ 1.55 V *vs.* Li corresponding to the two-phase insertion/extraction mechanism, (iii) zero-strain insertion that provides no volume change during charge–discharge process, (iv) no solid electrolyte interface (SEI) formation, (v) inexpensive raw materials, (vi) ease of preparation and (vii) eco-friendliness^4^,^20^,^21^. Pre-lithiated graphite has also been explored as anode for Li-HEC applications and has gained special attention due to its higher energy density and operating potential; however, tedious pre-lithiation and subsequent SEI formation is a major issue for scaling up of the graphitic anodes^4^,^5^. On the other hand, there is not much work reported on the development of supercapacitor components (carbonaceous electrodes) except activated carbon (AC) due to its unique features like high electrical conductivity and chemical stability^28^. Moreover, the reports based on carbon nanotubes (CNT)^17^,^22^ and activated graphene (AG)^29^ are available for Li-HEC applications. Utilization of bio-mass derived carbons is attractive in terms of specific capacity, ease of tailoring for desired applications and availability. Several biomass sources such as coconut-shell (CS), banana fibers, corn grain, sugar cane bagasse, apricot shell, sunflower seed shell, rice husk etc have been used to obtain ACs that have been effectively tuned for supercapacitor applications irrespective of the electrolyte medium^30^. Among them, CS derived carbons exhibits favorable electrochemical properties such as high specific capacity, good cycleability and availability^31^. Apart from the source material for carbon, the activation process *i.e.* chemical, CO_2_, steam, hydrothermal etc is very crucial to yield the high performance electro-active material. The AC obtained from hydrothermal treatment displays better structural, chemical and electrochemical properties compared rest of the activation process, particularly for bio-mass derived carbons^32^. In this regard, we have effectively used the hydrothermal activation process for CS along with chemical activation to obtain AC with high mesoporosity. AC are also additionally tailored by physical activation process, and subsequently employed as cathode active material in Li-HEC applications. The detailed synthesis of AC using hydrothermal treatment and electrochemical studies are discussed in the present paper.

## Results

**Figure 1:**
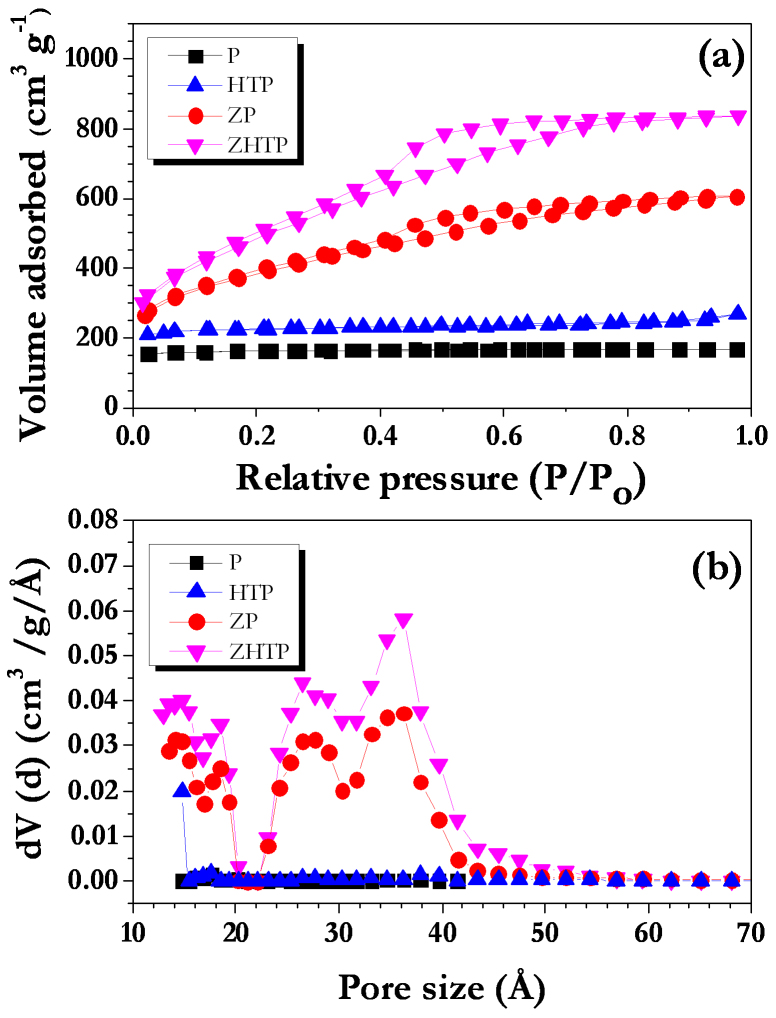
(a) Nitrogen adsorption isotherms of activated carbons synthesized at different experimental conditions, (b) Pore size distribution of activated carbons using DFT (Density Functional theory) model.

**Figure 2:**
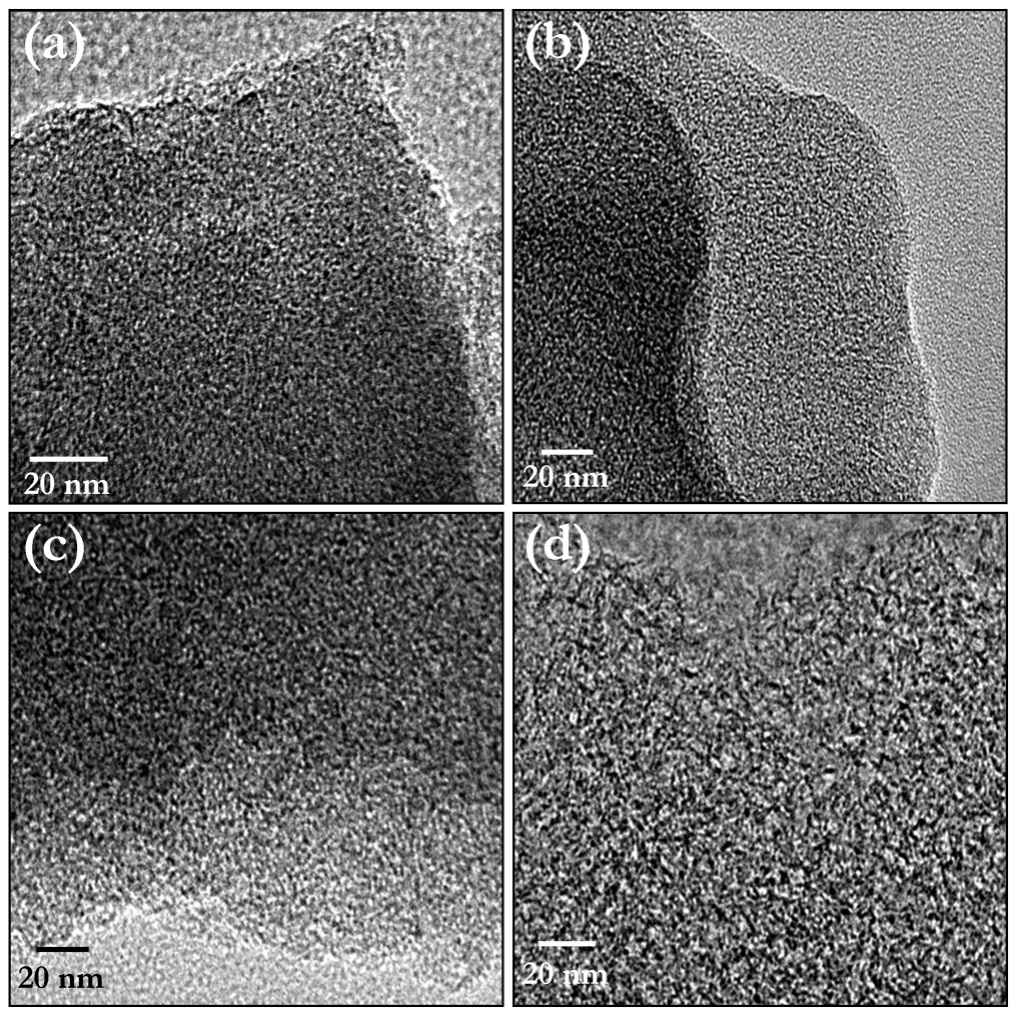
FE-TEM images ACs synthesized at different conditions: (a) P, (b) ZP (c) HTP and (d) ZHTP. Scale bar of 20 nm is fixed for all the four images.

Figure 1a shows nitrogen adsorption-desorption isotherms of ACs prepared at different conditions. P and HTP exhibit type I isotherm that is indicative of the presence of microporosity. ZP and ZHTP prepared with the ZnCl_2_ displayed a combination of both type II and type IV isotherms. In addition the hysteresis loops are more pronounced for both cases which suggest the formation of mesoporosity due to creation of additional pores and widening of small pores. Impregnation with ZnCl_2_ results in degradation of the cellulosic material present in the raw CS and carbonization together with dehydration which leads to the charring and aromatization of the carbon skeleton that eventually results in the formation of meso- and micro- porous of ACs. The porosity data (Table 1) clearly shows the important role of hydrothermal treatment along with chemical activation in increasing mesoporosity. ZnCl_2_ plays a vital role to enhance the pore formation process. First, the pore forming agent ZnCl_2_ dehydrates the CS. Pyrolysis at high temperature leads to the dehydration of carbon and creation of the micropores. Later, these micropores are getting widened and subsequently converted to mesopores in the presence of inert atmosphere^33^,^34^. The hydrothermal treatment step provides a critical advantage that enhances mesopore formation. The environment of elevated temperature and pressure provided during hydrothermal treatment facilitates better mass transfer and diffusion of ZnCl_2_ into the coconut shell which leads to more efficient chemical activation. As can be seen from Table 1, highest mesoporosity is obtained when the activation process includes use of ZnCl_2_ as the activating agent in the hydrothermal environment. This enhancement is also aided by the formation of more oxygen functional groups during hydrothermal treatment; functional groups present in the hydrothermally treated CS is favorable for chemical activation and also requires lesser activation energy for the burn off to generate porosity compared to direct pyrolysis. Apart from the surface area, pore size distribution is an important criterion for supercapacitors. Figure 1b shows the pore size distributions of ACs. As expected, pore size distribution for the AC treated with ZnCl_2_ falls in the mesoporous range. Both ZP and ZHTP exhibit two significant peaks at ~ 2.6 and ~ 3.4 nm which lie in the mesoporous range. In addition, mesopore content for ZHTP is ~ 60% compared to ~ 48% for ZP and ~ 20% for HTP. It is well known that presence of such mesopores will enhance the supercapacitance behavior of the carbonaceous electrode^35^. Therefore, enhanced electrochemical performance is expected for the chemically treated ACs in capacitance measurements. Figure 2 shows the FE-TEM images of ACs treated by different approaches. AC treated without ZnCl_2_ (P and HTP) have smoother textures, whereas morphologies of ZP and ZHTP show the consequence of mesopore generation in the form of increased roughness in the texture. Generally, such high surface area AC exhibits highly disordered nature which is clearly supported from the Raman analysis (Figure S1, supporting information) and consistent with literature^36^.

**Figure 3:**
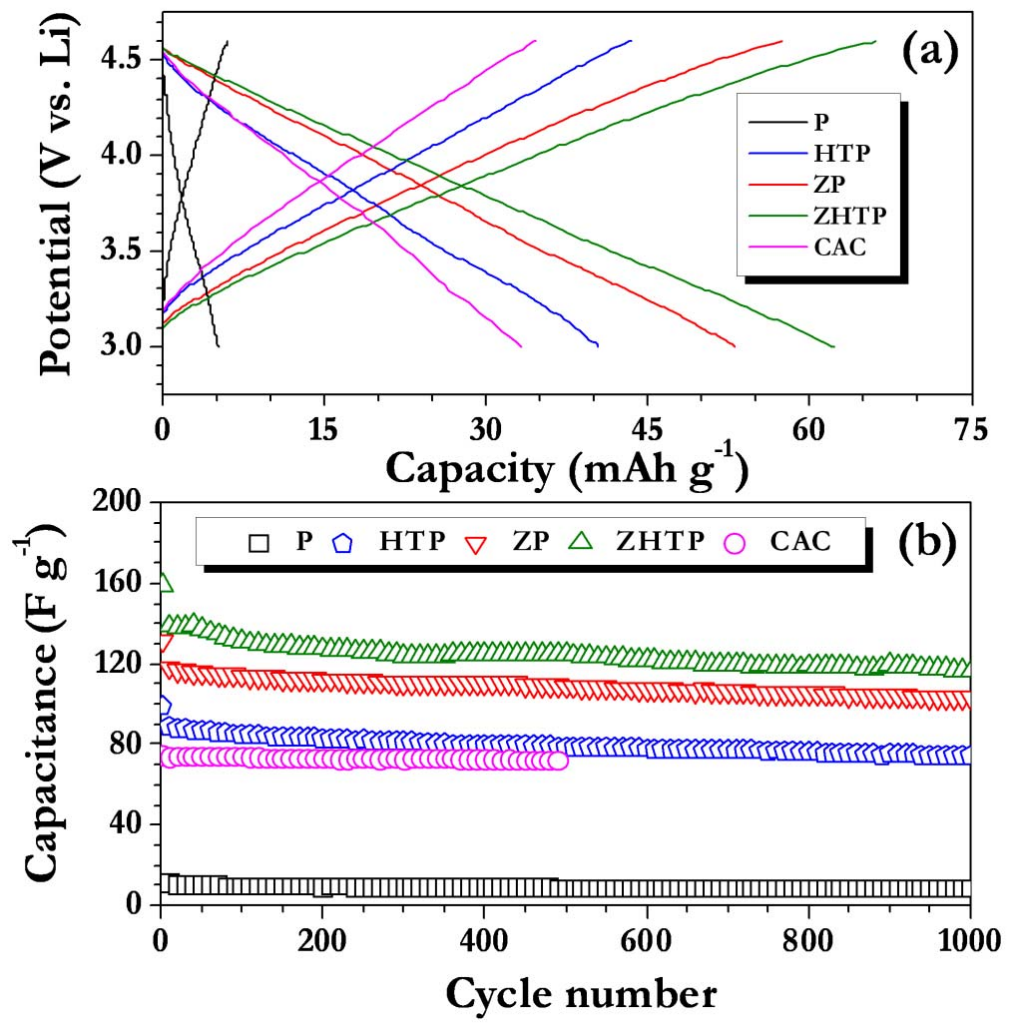
(a) Typical galvanostatic charge-discharge curves of various carbonaceous materials in half-cell or single electrode configuration between 3–4.6 V *vs.* Li at current density of 100 mA g^−1^, in which metallic lithium act as counter and reference electrode, and (b) Plot of specific discharge capacitance *vs.* cycle number for above mentioned cells in ambient conditions. Data points are collected after every 10 cycles.

Single electrode performance of the carbonaceous material is very crucial to employ them in Li-HEC along with insertion type anode Li_4_Ti_5_O_12_. In the conventional two electrode symmetric supercapacitor configuration, the applied potential is equally split between the two electrodes, whereas the Li-HEC configuration contains two different electrodes which undergo two different storage mechanisms. Therefore, the applied voltage will split between the electrodes depending on the capacitance of each electrode. Hence, mass balance between the electrodes is necessary to achieve higher energy density while employing different kinds of materials which undergo two different energy storage mechanisms *i.e.* reversible Faradaic (Li-insertion type electrode) and reversible non-Faradaic process (supercapacitor component)^37^. Half-cell configuration (Li/AC) of ACs are tested between open circuit voltage (OCV) to decomposition potential of the conventional carbonate based electrolyte solution (~4.6 V *vs.* Li) at current density of 100 mA g^−1^. Moreover, such half-cell configuration is considered as three electrode or single electrode configuration to elucidate the supercapacitive properties of the individual component. During electrochemical reaction, the test electrode undergoes PF_6_^−^ anion adsorption/de-sorption process, since metallic lithium acts as both counter and reference electrodes. Typical galvanostatic charge-discharge profiles are illustrated in Figure 3. A linear increase in potential with respect to time is evidenced irrespective of the samples tested. Such linear variation clearly suggests the perfect adsorption and de-sorption process of PF_6_^−^ anions and subsequent double layer formation across the electrode/electrolyte interface^28^,^37^. The commercially available AC was also tested in half-cell configuration and compared along with CS derived AC under the same current rates. The half-cell delivered initial reversible capacities of 5, 44, 58, 71 and 33 mAh g^−1^ for P, HTP, ZP, ZHTP and CAC, respectively. The observed discharge capacities can be converted to specific capacitances according to the following relation that is valid for linear variation of voltage with time^2^. 
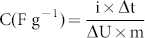
where, *I* is applied current (A), Δ*t* is discharge time (s), *m* weight of the active material (g) and ΔU is testing window (*i.e.* potential difference) of the aforementioned half-cell configuration (mV, 1600 mV). Initial specific discharge capacitance of ~ 11, ~ 99, ~ 132, ~ 159, and ~ 74 F g^−1^ are obtained for P, HTP, ZP, ZHTP and CAC, respectively. It is worth noting that increase in mesoporosity results the increase in specific capacitance and is consistent with the results of Simon and Gogosti^35^. Plot of specific discharge capacitance *vs.* cycle number is given in Figure 3b. Very stable specific capacitance profiles are observed during prolonged cycling for all samples, except for a small drop in the second cycle. Further, the observed capacitance value is linear with specific surface area which is also consistent with the literature^28^. Since increase in surface area and mesoporosity leads to the accommodation of more anions, the AC is able to provide higher specific capacitance. ZP and ZHTP delivered specific discharge capacitances that are almost two times higher than those obtained using CAC electrodes (Figure 3). Therefore, a similar enhancement in the performance is expected in Li-HEC configuration when coupled with spinel phase Li_4_Ti_5_O_12_ anode. Furthermore, HTP also delivers higher reversible adsorption/de-sorption behavior than commercial AC powders. This clearly suggests that the tailoring of carbonaceous materials with appropriate pore sizes is very crucial to yield high performance materials. The enhancement of such AC are mainly because of phase purity (Table T1) and high surface area with mesopores, which are required for easy access of the electrolyte solution and thereby enables facile adsorption/de-sorption of PF_6_^−^ anions especially at high current rates^38^. Based on the electrochemical performance of both AC cathodes derived from CS (Li/AC) and spinel phase Li_4_Ti_5_O_12_ (Li/Li_4_Ti_5_O_12_, Figure S2, supporting information) in half-cell configuration under same current densities, the mass loading optimized and adjusted by the ratio of anode to cathode is 1:3.88, 1:2.91 and 1:2.41 for samples HTP, ZP, and ZHTP, respectively. However, the performance of sample P is found to be very inferior compared to the rest of the ACs; therefore, the powder is not been tested in Li-HEC configuration.

## Discussion

**Figure 4:**
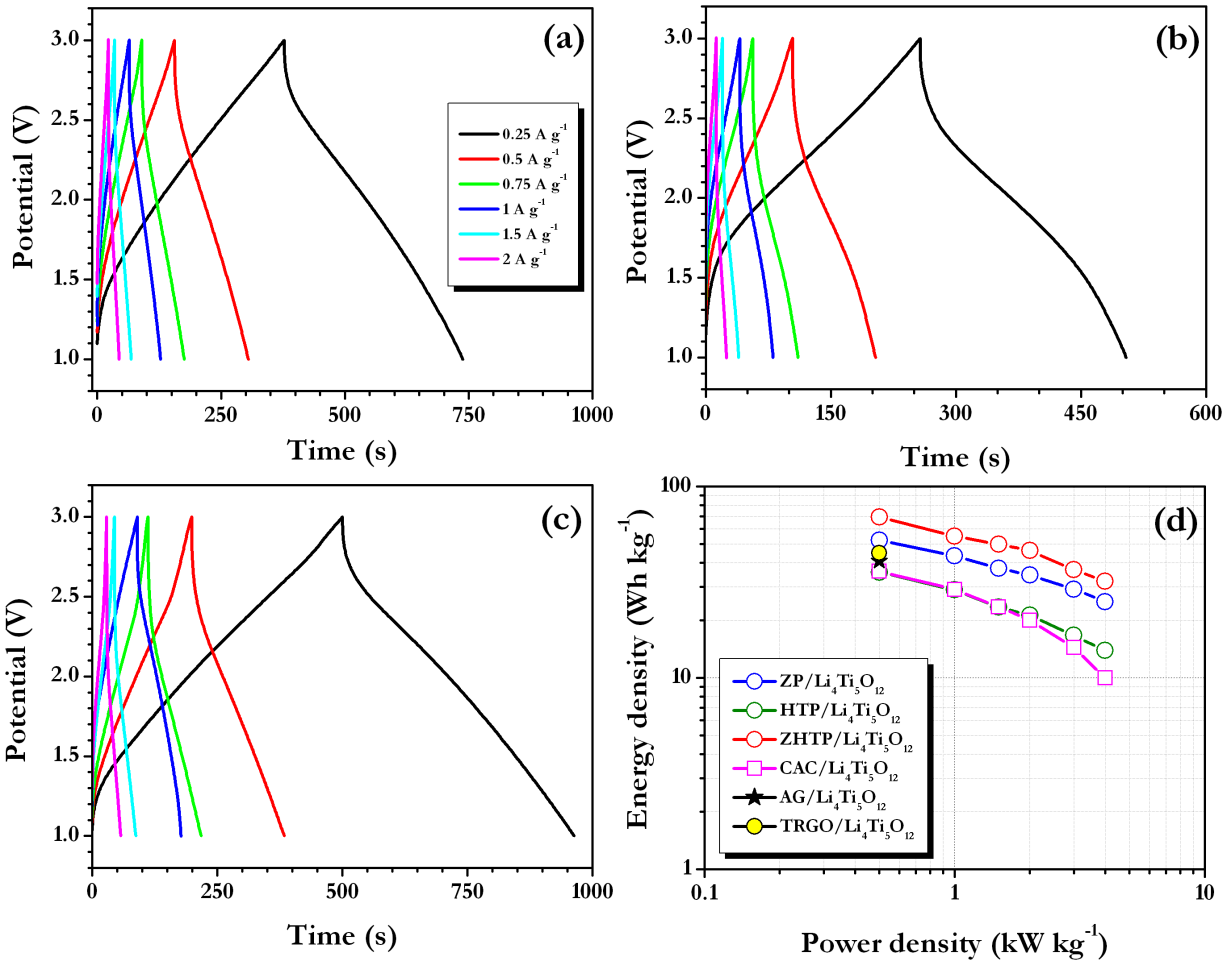
Galvanostatic charge-discharge curves of Li-HEC comprising spinel phase Li_4_Ti_5_O_12_ anode and coconut shell derived carbons as cathode (a) ZP (b) HTP and (c) ZHTP, and (d) Ragone plot of Li-HEC along with CAC cathode and activated graphene (AG) based Li-HEC by Stoller *et al.*^29^, trigol reduced graphene oxide (TRGO) based Li-HEC by Aravindan *et al.*^39^.

**Figure 5:**
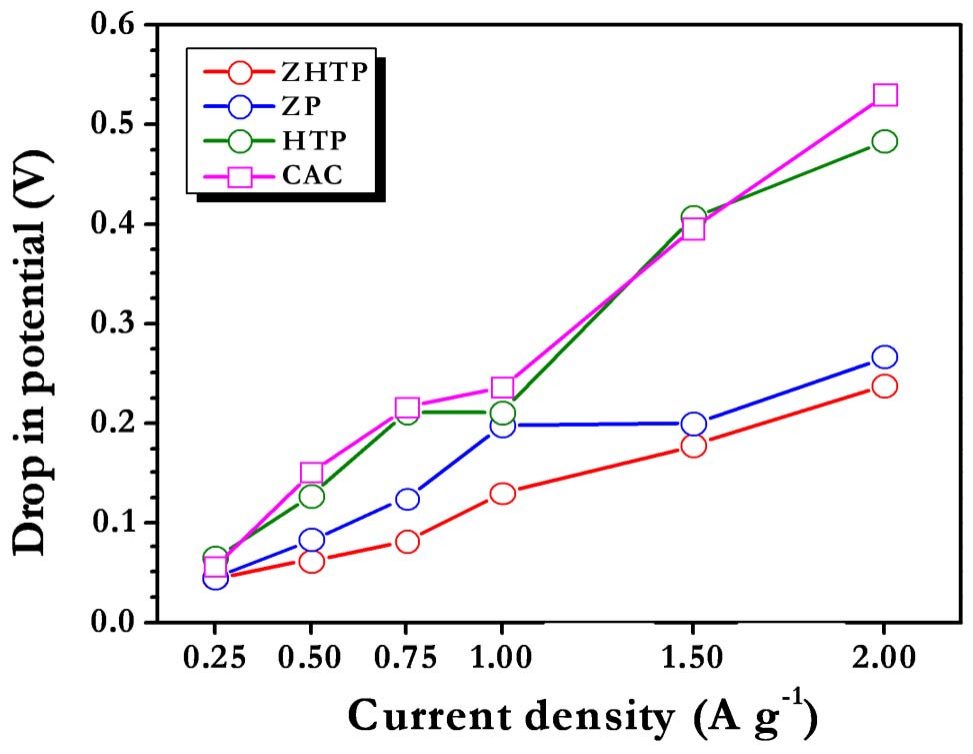
Plot of potential drop (IR drop) *vs.* applied current density.

Figure 4a–c shows the galvanostatic cycling profiles of Li-HEC comprising various carbonaceous cathodes recorded between 1–3 V for 100% depth of discharge (DOD) at various applied current densities at ambient conditions. Applied current rates are calculated based on the total mass of both anode and cathode. During the charging process, spinel phase Li_4_Ti_5_O_12_ anode undergoes reversible Li-insertion whereas the carbonaceous cathode involves electric double layer formation with PF_6_^−^ anions across the electrode/electrolyte interface to provide necessary power density. The said reaction is reversed during subsequent discharge. The reaction mechanism is clearly validated from the cyclic voltammetric measurements (Figure S3). Similar to the results for the single electrode configuration, ZHTP displayed longer discharge times compared to ZP and HTP in Li-HEC assembly. Further, the observed values are higher than those obtained from CAC (Figure S4, supporting information). As mentioned earlier, tailoring of AC from CS with high surface area and mesoporous nature is the main reason for such increase in discharge time. As a result, significant enhancements in the energy densities are noted as evidenced from Figure 4d (Ragone plot). The average energy and power densities are calculated from the formulae given in the literature^16^,^17^,^18^,^23^,^24^,^25^,^27^. Briefly, specific energy (E_SP_) and power densities (P_SP_) are calculated using following relations: P_SP_ = (U · i/m) and E_SP_ = (P_SP_ · t), where U = (U_max_ + U_min_)/2, U_max_ and U_min_ are respectively the potential at beginning of discharge and at the end of discharge curves of galvanostatic cycle. The Li-HEC comprising ZHTP delivered a high energy density of ~ 69 Wh kg^−1^ compared to rest of the ACs tested such as ZP (~52 Wh kg^−1^), HTP (~36 Wh kg^−1^) and CAC (~36 Wh kg^−1^). Apart from the dramatic two fold increase in energy density, the enhancement in power density of the Li-HEC system comprising CS-ZHTP is noteworthy. The observed energy densities are much higher than to drive HEV (~7.5–8.3 Wh kg^−1^), but still further enhancement are required to drive PHEV (~57–97 Wh kg^−1^) and EV (min. 150 Wh kg^−1^)^7^. In addition, present results are much higher than the those reported by Stoller *et al.*^29^, in which KOH treated microwave exfoliated graphite oxide was used as cathode active material along with Li_4_Ti_5_O_12_ anode to deliver a maximum energy density of 40.8 Wh kg^−1^. Recently, we reported the performance of trigol reduced graphene oxide nanosheets as cathode active material in Li-HEC assembly with Li_4_Ti_5_O_12_ anode and such assembly delivered a maximum energy density of ~ 45 Wh kg^−1^^39^. Further, Li-HEC is also capable of driving miniature electronic applications for example; various light emitting diodes (LED) are tested with charged Li-HEC and shown in figure S5. This clearly suggests, Li-HEC not only drive the high power applications likely HEV and also can able to power small scale electronic devices. To the best of our knowledge, the observed value is one of the best values reported in Li-HEC applications, other than graphitic anodes^5^,^29^. The present study clearly shows that CS derived AC with appropriate mesoporosity and specific surface area can be used as prospective electrode material for high performance Li-HEC applications. Internal resistance of the Li-HEC plays the vital role to enhance the power density of the system irrespective of the electrolyte medium used. In this line, variation in the potential drop *vs.* applied current densities is measured and given in Figure 5. The increase in resistance with respect to applied current densities is almost linear. A very small IR drop is observed for ZHTP compared to rest of the samples particularly in high current operations, which certainly promotes the enhancement of power density without compromising energy density. This is a direct measurement of resistance offered by the electro-active material during the discharge process of Li-HEC.

**Figure 6:**
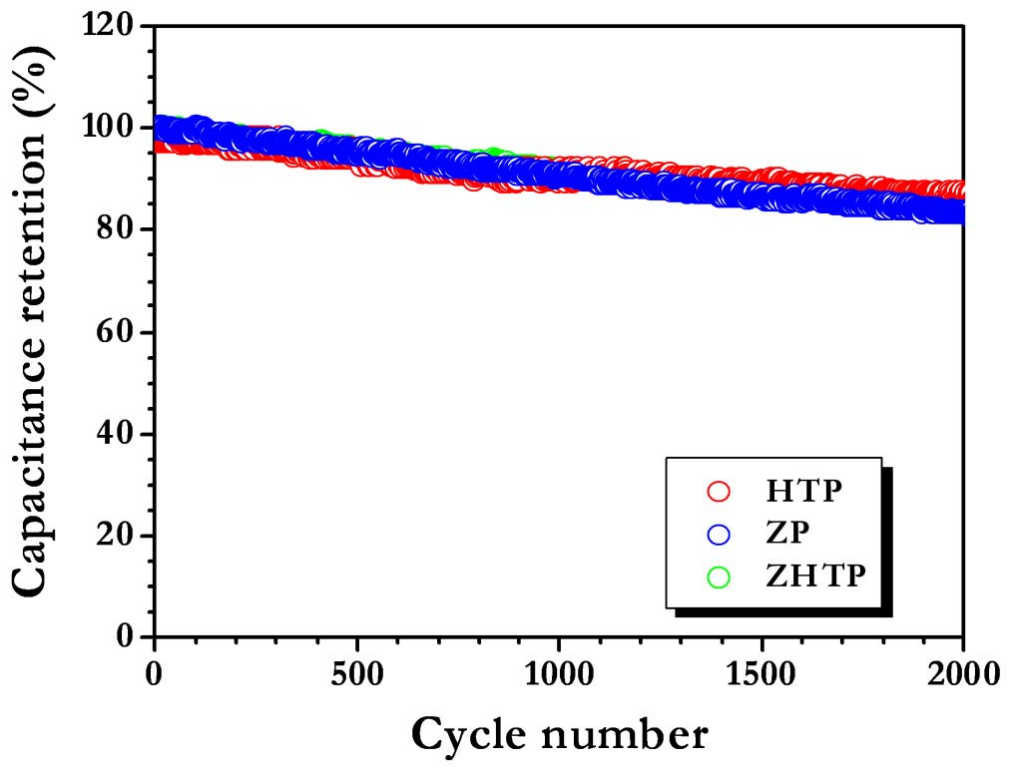
(Normalized) Cycling profiles of Li-HEC comprising high surface area carbonaceous anode materials between 1–3 V at current density of 1.5 A g^−1^.

Cycleability is another important criterion for evaluation of materials as cathode in Li-HEC applications. Duplicate cells were made from CS AC along with Li_4_Ti_5_O_12_ anode and cycled at a current rate of 1.5 A g^−1^ for 100% depth of discharge (Figure 6). Li-HEC rendered good cycleability for 2000 cycles and retained the initial discharge capacitance to the extent of ~ 85, ~ 85 and ~ 83% for ZHTP, HTP and ZP, respectively. The small capacitance loss during cycling is mainly due to the intrinsic nature of the commercial Li_4_Ti_5_O_12_ insertion type anode (Figure S2), whereas very stable cycleability is noted for carbonaceous cathodes (Figure 3). Moreover, this fading issue can be alleviated by adopting highly conducting networks such as CNT or carbon nanofibers as suggested by Naoi and co-workers^4^,^5^,^6^,^20^,^21^. As described above, this study certainly provides a new platform to employ high surface area AC from bio-masses for real time energy storage applications, preferably in Li-HEC to realize high energy and power densities. Similar procedures can be easily extended to other bio-masses such as banana fibers, corn grain, sugar cane bagasse, apricot shell, sunflower seed shell, rice husk etc. to explore as prospective electrode materials for high energy density electrochemical energy storage devices.

In summary, high surface area mesoporous AC carbons were derived from CS. The AC were tailored by adopting various approaches to yield high performance supercapacitor components and delivered the maximum specific capacitance of ~ 159 F g^−1^ in non-aqueous medium. Li-HEC was fabricated with commercial Li_4_Ti_5_O_12_ anode under the optimized mass loadings based on the single electrode performance of each component. AC (CS-ZHTP) comprising high mesoporosity (60% mesoporous volume) with surface area of 1652 m^2^ g^−1^ delivered extremely high energy density of ~ 69 Wh kg^−1^. The observed values were much higher than reported values elsewhere by using either graphene or AC obtained from other sources as cathode active material in Li-HEC configuration. Further, this approach can be easily transferred to other bio-mass precursors and engineered to fabricate high performance electro-active materials for energy storage applications.

## Methods

Coconut (*Cocos nucifera*) shells (CS) were obtained from Malaysia. Analytical grade chemicals such as zinc chloride (ZnCl_2_) and sodium hydroxide were procured from Merck and Sigma-Aldrich, respectively and used as such. The commercially available AC was obtained from Norit, (The Netherlands) with surface area of 818 m^2^ g^−1^. The fibers on the shell were trimmed and the shells were dried at 105°C for 24 h; the dried shells were ground in a commercial lab blender and sieved to extract 10–20 mesh.

### Synthesis of activated carbons (AC)

ACs was processed by chemical and physical activation procedures, successively. For the chemical activation process, ZnCl_2_ was employed as pore forming agent in the desired weight ratio with respect to CS granules and CO_2_ was employed for physical activation step. First, the dried CS granules were mixed with ZnCl_2_ solution (5 g of CS granules in 30 mL water) in the predetermined ZnCl_2_ to CS granules ratio and dried at 105°C for 12 h. Then, the samples were loaded into alumina boats and heated to 800°C in a Carbolite horizontal tube furnace. Temperature was ramped at a rate of 10°C min^−1^ in the presence of N_2_ at a flow rate of 50 mL min^−1^. After the temperature reached 800°C, the N_2_ gas was changed to CO_2_ at a flow rate of 40 mL min^−1^ for 2 h. Finally, the furnace was cooled to room temperature in the presence of N_2_ at a flow rate of 50 mL min^−1^. The products were transferred to a beaker containing 0.1 M of HCl solution, stirred for 30 min and washed with distilled water until the pH reached 6. Finally, the washed AC was dried at 105°C for 24 h and then used for further experiments (P).

### Hydrothermal treatment

A Parr 4843 autoclave was charged with 25 g of CS granules and 150 mL water with the predetermined ZnCl_2_: CS granules ratio (2:1). The samples were held at 275°C for 20 min at the self-generated pressure of 53 bar. The hydrothermally treated products were cooled down to room temperature and were dried at 105°C for 12 h.

Four types of AC were prepared and evaluated as cathode active material for Li-HEC applications. The AC obtained from pyrolyzed raw CS granules (i.e. CS granules were ramped upto 800°C in presence of N_2_ flow. After reaching 800°C, the N_2_ flow was switched to CO_2_ atmosphere and maintained for 2 h) was used as first sample (hereafter abbreviated as ‘P’). The second one (ZP) was obtained by soaking CS granules in ZnCl_2_ solution (ZnCl_2_:CS granules ratio 2:1) at 105°C for 12 h and ramped upto 800°C in presence of N_2_ flow. After reaching 800°C, the N_2_ flow was switched to CO_2_ flow and maintained for 2 h. The third sample (HTP) was prepared using CS granules treated in the hydrothermal environment in the absence of ZnCl_2_ and pyrolyzed at 800°C in the presence of N_2_ and CO_2_ as discussed earlier. Finally, the fourth sample (ZHTP) was obtained by treating CS granules with ZnCl_2_ (ZnCl_2_:CS granules ratio 2:1) in the hydrothermal reactor and pyrolyzed at 800°C in the presence of N_2_ and CO_2_ flows as discussed earlier. Commercially available AC is named as ‘CAC’.

### Characterization

Elemental analysis was performed using an elemental analyzer (Vario Macro Cube, Elementar, Germany). Adsorption-desorption isotherms, surface area and pore volume determination of the adsorbents were obtained and analyzed using gas sorption analyzer (Nova-3000 Series, Quantachrome). The BET (Brauner-Emmett-Teller) equation was employed for surface area measurement, while DFT (Density Functional Theory) method was used for obtaining pore size distribution. The surface morphology of ACs was studied with field emission transmission electron microscopy (FE-TEM) (JEOL JEM-2100F), using an accelerating voltage of 200 kV.

### Electrochemical studies

All the electrochemical characterizations were carried out in standard two electrode coin cell (CR 2016) configuration. Composite electrodes were formulated with accurately weighed (10 mg) active material (AC or Li_4_Ti_5_O_12_), 1.5 mg of conductive additive (super P) and 1.5 mg of binder (Teflonized acetylene black, TAB-2) for half-cell assembly (Li/AC or Li/Li_4_Ti_5_O_12_) using ethanol as solvent. Then the mixture was pressed over a 200 mm^2^ stainless steel mesh, which served as current collector. The composite electrodes were dried at 60°C overnight prior to cell assembly in an Ar filled glove box. Test electrodes in the coin cells were separated by a microporous glass fiber separator (Whatman, Cat. No. 1825-047); 1 M LiPF_6_ in ethylene carbonate (EC)/di-methyl carbonate (DMC) (1:1 wt.%, Selectipur 30) mixture was used as electrolyte solution. For half-cell measurements, metallic lithium was used as the counter and reference electrode. In the case of Li-HEC configuration, AC acted as cathode and commercially available spinel phase Li_4_Ti_5_O_12_ (Sigma-Aldrich, USA) served as anode in non-aqueous medium. Galvanostatic charge-discharge profiles were performed in both half and full-cell configurations using Arbin BT2000 battery tester at ambient temperature conditions.

## Author Contributions

V.A. and S.J. designed the project and wrote the manuscript. A.J. and V.A. carried out the experiments and electrochemical studies. P.S.K. helped to record TEM images. R.B., S.R., M.S. and M.P.S. involved in the discussion and finalizing the manuscript.

## Supplementary Material

Supplementary Information Supplementary information

## Figures and Tables

**Table 1:** BET Surface area and mesoporosity of synthesized ACs at different conditions

Sample	BET Surface area (m^2^ g^−1^)	Mesopore surface area (m^2^ g^−1^)	Total pore volume (mL g^−1^)	Mesopore volume (mL m^2^ g^−1^)	Yield (%)	Area_me_/Area_t_ (%)	V_me_/V_t_ (%)
**P**	533 ± 29	10 ± 2.85	0.27 ± 0.12	0.011 ± 0.01	25 ± 0.84	1.8	4.1
**ZP**	1421 ± 75	383 ± 17	0.98 ± 0.04	0.468 ± 0.02	23.5 ± 2.7	26.9	47.7
**HTP**	712 ± 2	44 ± 5.1	0.406 ± 0.01	0.081 ± 0.01	24.2 ± 2.4	6.18	19.9
**ZHTP**	1652 ± 132	640 ± 53	1.29 ± 0.01	0.768 ± 0.1	22 ± 2.3	38.7	59.5

Area_me_: Mesoporous surface area, Area_t_: Total surface area, V_me_: Mesoporous volume and V_t_: total volume.
