# Supplementary material for: Activated carbons derived from coconut shells as high energy density cathode material for Li-ion capacitors
Source: Sci Rep. 2013 Oct 21;3:3002. doi: 10.1038/srep03002 (PMC3801125; doi:10.1038/srep03002)
Supplement: Supplementary Information [file srep03002-s1.pdf]

Supplementary information

Activated carbons derived from coconut shells as high energy density cathode material for Li-ion capacitors

Akshay Jain[a], Vanchiappan Aravindan[b*], Sundaramurthy Jayaraman[c], Palaniswamy Suresh Kumar[c], Rajasekhar Balasubramanian[d], Seeram Ramakrishna[c], Srinivasan Madhavi[b,e*], and M. P. Srinivasan[c]

[a] Department of Chemical and Biomolecular Engineering, National University of Singapore, Singapore 117576

[b] Energy Research Institute @ NTU (ERI@N), Nanyang Technological University, Singapore 637553, E-mail: aravind_van@yahoo.com

[c] Department of Mechanical Engineering, National University of Singapore, Singapore 117576,

[d] Department of Civil and Environmental Engineering, National University of Singapore, Singapore 117576

[e] School of Materials Science and Engineering, Nanyang Technological University, Singapore 639798. E-mail: Madhavi@ntu.edu.sg

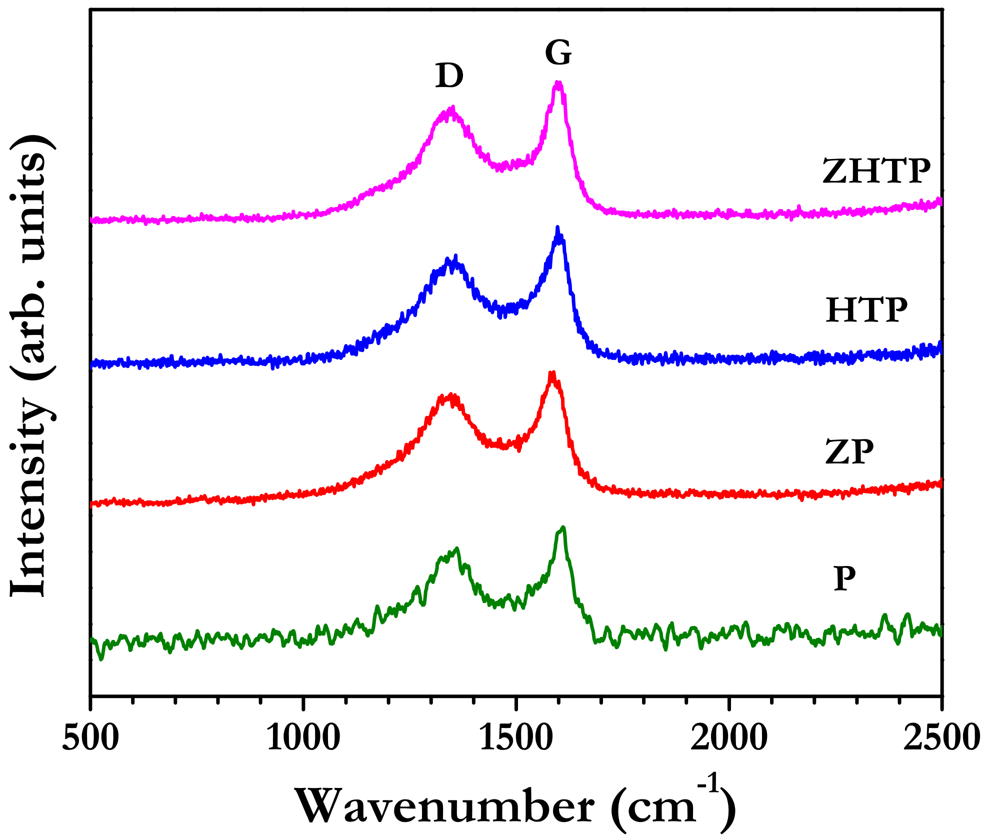

Figure S1. Raman spectra of high surface area carbonaceous materials derived from the coconut shells

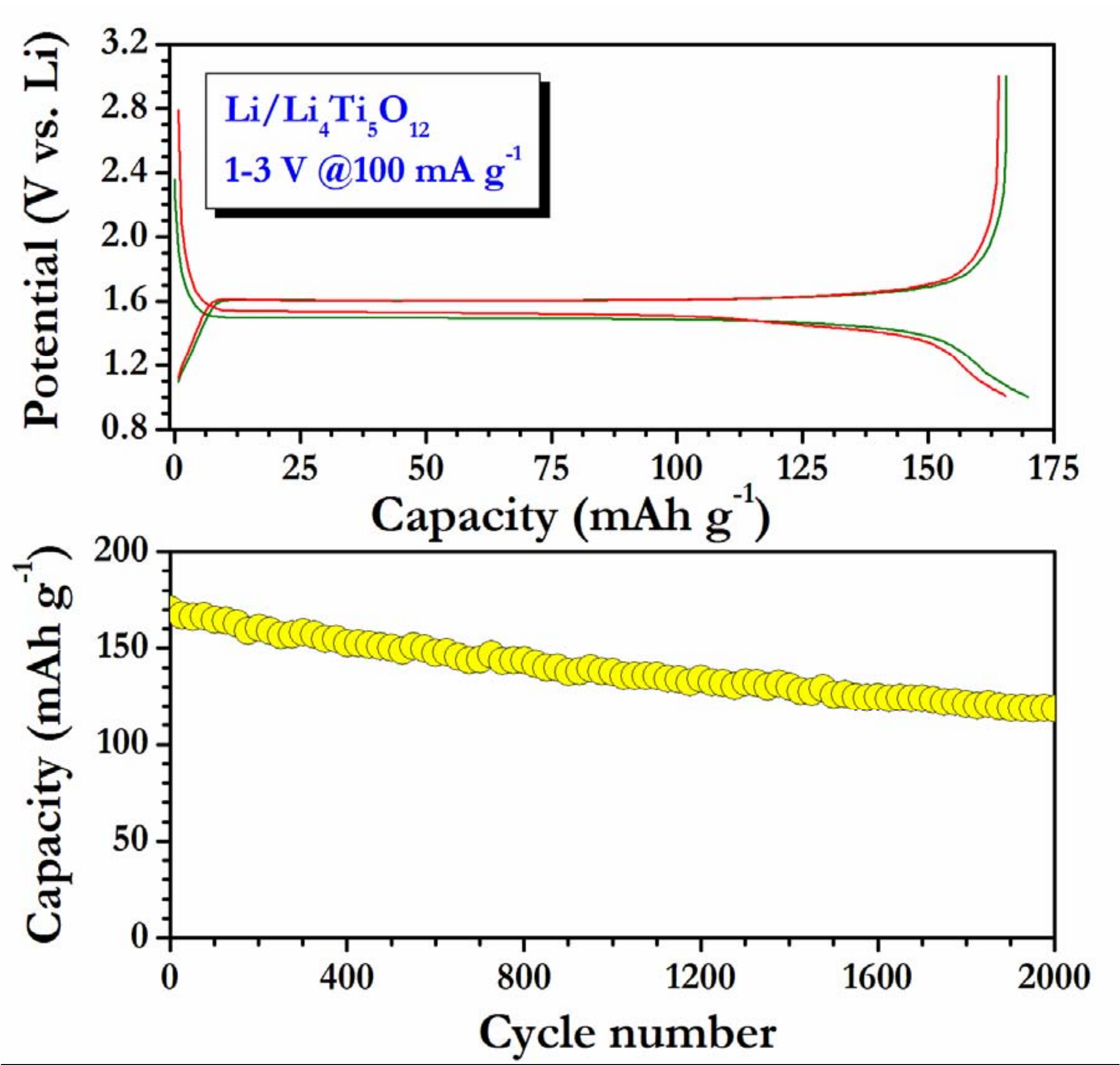

Figure S2. Galvanostatic charge-discharge curves of Li/$Li_4Ti_5O_{12}$ (Aldrich, USA) half-cells cycled between 1-3 V at constant current density of 100 mA g^{-1}, and (b) plot of capacity *vs.* cycle number

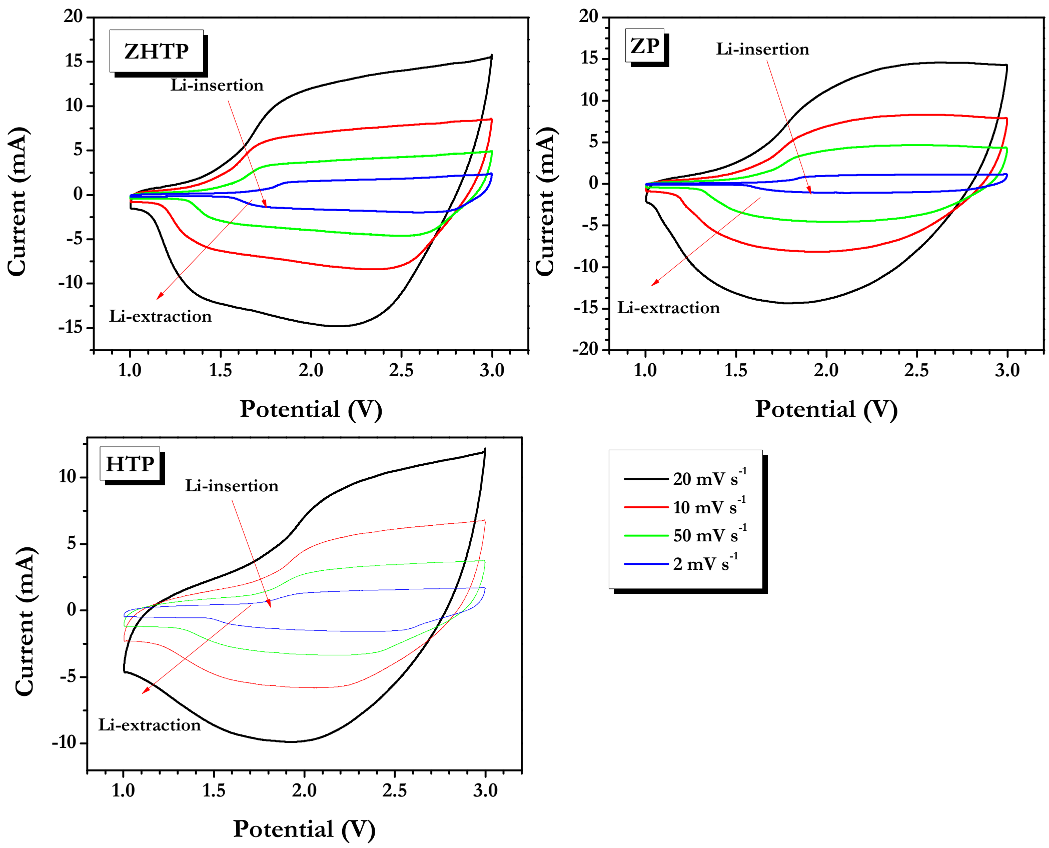

Figure S3. Cyclic voltammetric traces of Li-HEC comprising various carbonaceous materials with insertion type $Li_4Ti_5O_{12}$ anode. It is evident from CV profiles, above/below 1.5 V a drastic increase/decrease in current response is observed for all the three cases which indicate the Li-insertion/extraction in to/from spinel lattice for all the three configurations. Similarly, a prominent current response is noted during both anodic and cathodic scan which indicates the formation of electric double layer across the carbonaceous cathode and electrolyte interface. The observed CV traces are consistent with Leng *et al.* Nano Res. **2013**, 6, 581 –592.

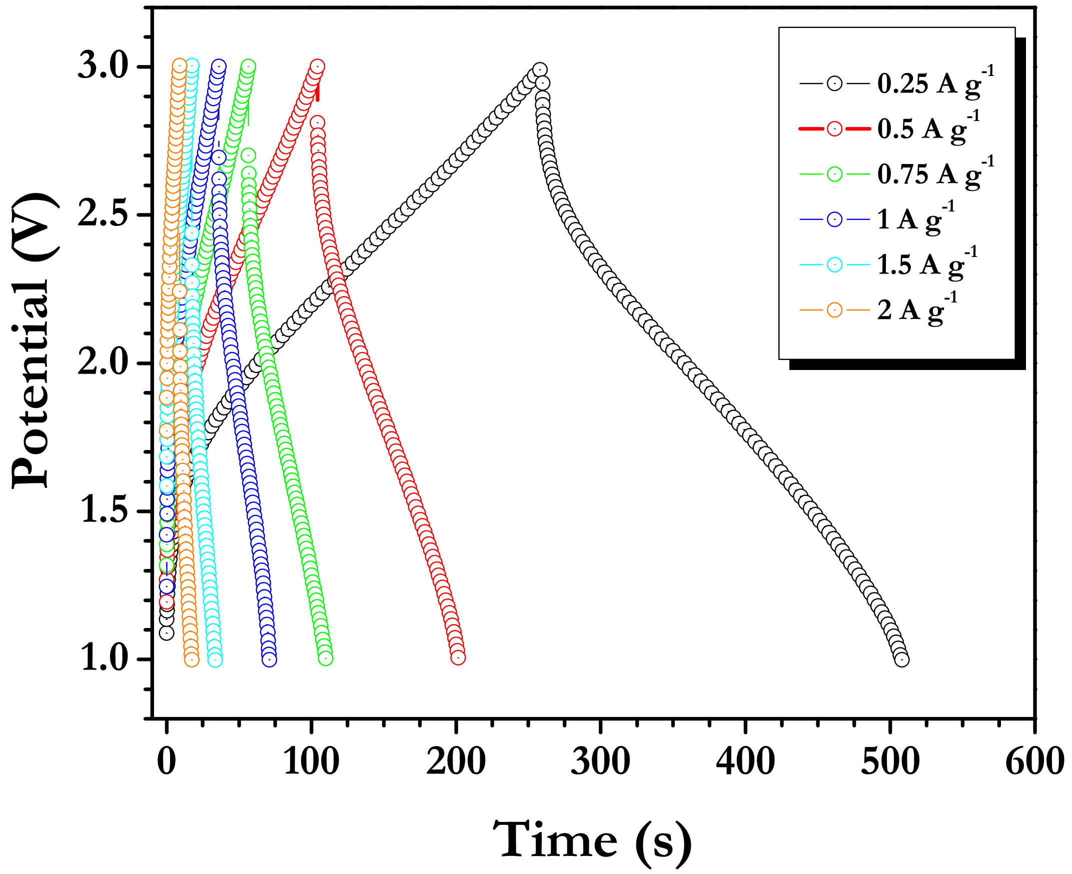

Figure S4. Typical galvanostatic charge-discharge curves of CAC cathode with $Li_4Ti_5O_{12}$ anode in Li-HEC configuration

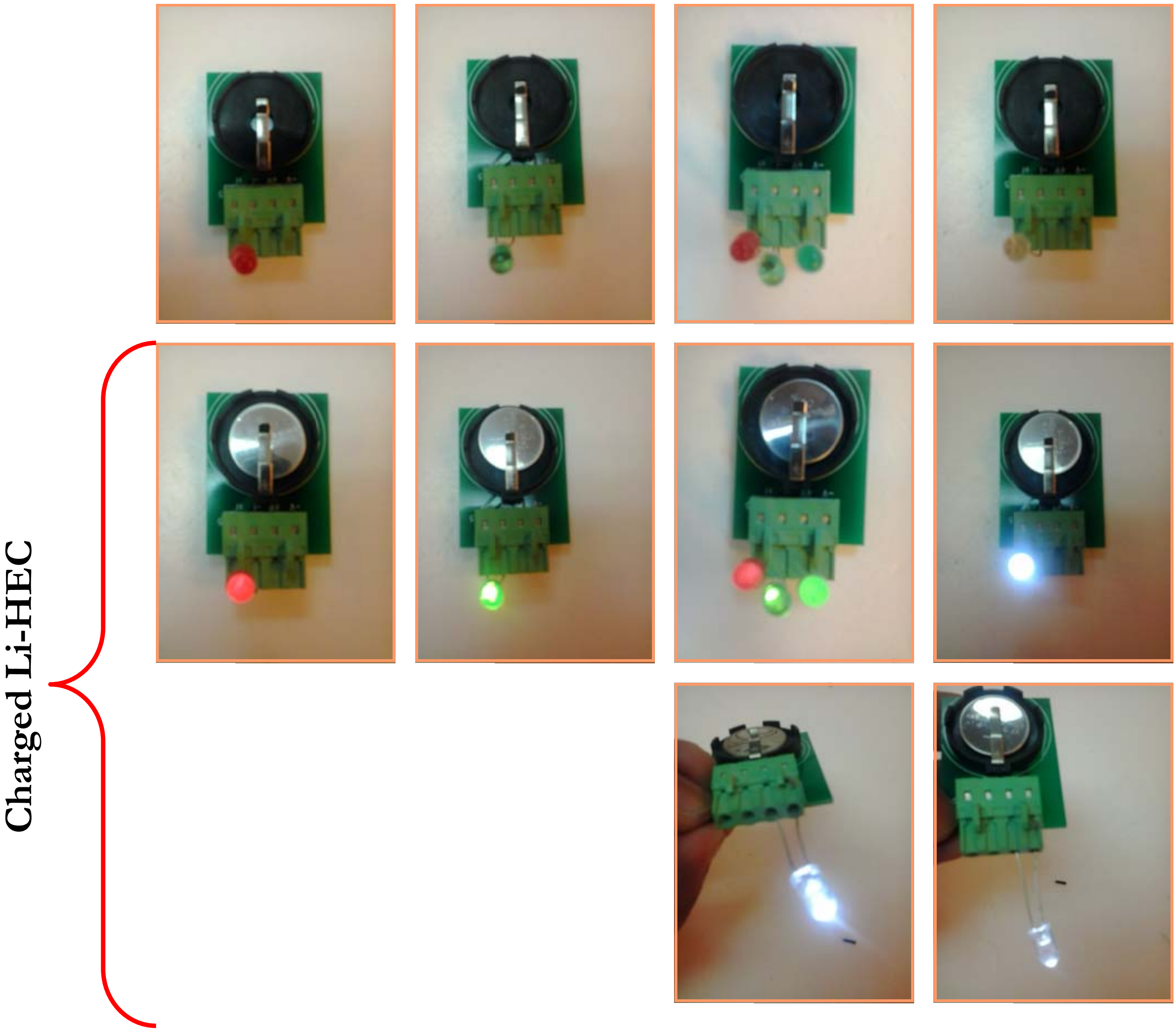

Figure S5. Demonstration of various LEDs powered by Li-HEC (ZHTP/$Li_4Ti_5O_{12}$). Initially, Li-HEC was charged to 3 V (at current density 100 mA g^{-1}) and placed in the coin-cell holder to drive LEDs.

Table T1. CHNS elemental analysis of synthesized ACs

Sample	P	ZP	HTP	ZHTP
%N	1.78	1.72	2.83	1.27
%C	89.57	86.55	81.94	91.53
%H	1.7	1.52	1.57	1.59
%S	0.78	0.52	0.43	0.51
